# Testing an online measure of portion size selection: a pilot study concerned with the measurement of ideal portion size

**DOI:** 10.1186/s40814-021-00908-x

**Published:** 2021-09-17

**Authors:** Rochelle Embling, Michelle D. Lee, Menna Price, Laura L. Wilkinson

**Affiliations:** Department of Psychology, College of Human and Health Sciences, Swansea University, Swansea, SA2 8PP UK

**Keywords:** Portion size, Meal size, Expected satiety, Expected satiation, Online, Survey, Pilot

## Abstract

**Background:**

Portion size is known to be a key driver of food intake. As consumed portions are often pre-planned, ‘ideal portion size’—an individual’s preferred meal size selected prior to eating—has been identified as a strong predictor of actual consumption. However, assessments of ideal portion size have predominantly relied on laboratory-based computer tasks, limiting use online. Therefore, this cross-sectional study sought to pilot test the validity of a web-based tool to measure ideal portion size.

**Methods:**

In an online study (*N* = 48), participants responded to images of a range of foods. Each food was photographed in a series of different portions and loaded into an ‘image carousel’ that would allow participants to change the size of the displayed portion by moving a slider left-to-right. Using this image carousel, participants selected their ideal portion size. They also completed measures of expected satiety and expected satiation and self-reported their age and body mass index (BMI). A non-parametric correlation matrix was used to explore associations between ideal portion size and identified predictors of food intake.

**Results:**

Supporting convergent validity of this measure, ideal portion size was significantly correlated with expected satiety (*r*_s_ = .480) and expected satiation (*r*_s_ = −.310) after controlling for effects of baseline hunger and fullness, consistent with past research. Similarly, supporting divergent validity of this measure, ideal portion size was not significantly correlated with age (*r*_s_ = −.032) or BMI (*r*_s_ = −.111,).

**Conclusions:**

Pilot results support the validity of this web-based portion size selection tool used to measure ideal portion size, though further research is needed to validate use with comparisons to actual food intake.

**Supplementary Information:**

The online version contains supplementary material available at 10.1186/s40814-021-00908-x.

## Key messages

- *What uncertainties existed regarding the feasibility?* The feasibility of a ‘continuous-scale’ assessment of portion size selection had yet to be tested for use in an online web-based survey (consisting of many images presented consecutively). In addition, the convergent and divergent validity of such a tool was unknown.
- *What are the key feasibility findings?* Forty-eight participants completed the study and were included in data analyses. Results suggested that participants’ ideal portion size—selected using a novel online portion selection tool—significantly correlated with expected satiety (as a well-established driver of ideal portion size; convergent validity) and did not significantly correlate with age or BMI (as factors that have previously been found to lack a relationship with ideal portion size; divergent validity).
- *What are the implications of the feasibility findings for the design of the main study?* Results support the validity of our web-based portion size selection tool to measure ideal portion size. Further research is needed to validate use with comparisons to actual food intake, and future studies should consider strategies to control for potential methodological limitations of measuring portion size in an online setting.

## Introduction

‘Portion size’ is known to have an important influence on food intake. Larger portions encourage greater meal consumption, with people consuming 35% more food on average when offered portions that are doubled in size [1]]. Across studies, this effect has been shown to occur for multiple food types, in different meal settings, and irrespective of individual characteristics (e.g. age, body mass index [BMI], and gender) [2–6]. In light of increasing portion sizes in the food environment [7, 8], portion size is one factor that has been highlighted as a potential contributing factor to overweight and obesity [9, 10].

Consumer decisions around portion size are likely to occur *before* eating when planning meals [11]. In a large online study, most individuals self-reported that they ‘cleared’ all foods on their plate, that they did so both when portions were self-selected and when offered fixed servings, and plate-clearing was found to have occurred most frequently when meals were planned [12]. Similar results have been found when measuring intended and consumed portions in the laboratory. For example, Robinson et al. [13] reported little difference between planned (74.2% to be eaten) and actually consumed (71.3% eaten) meal size when participants were served an ice-cream dessert. As such, ‘ideal portion size’—an individual’s preferred portion of a food that is selected prior to a meal—has been identified as a strong predictor of actual food intake [14], and has been supported as a measure that captures expected differences in consumption [e.g. for clinical patients with obesity before and after treatment, for low intake vs. high intake decision contexts, and for high energy-dense vs. low energy-dense foods [15]]. In light of the COVID-19 pandemic and the necessary suspension of face-to-face testing, the aim of this study was to pilot test the validity of an online tool to measure participants’ ideal portion size.

To measure ideal portion size, studies typically use a computer-based task [for the original task, see [16]]. Participants are presented with an array of photographs for each food item, with each image representing an incremental increase (or decrease) in portion size. These photographs are presented consecutively onscreen so that keyboard responses give rise to an ‘animated’ effect, as the displayed portion of food appears to grow or shrink with each keyboard press, and participants are asked to select the portion size that they would most like to consume in a specified context. Using this measure, past studies have shown that ideal portion size is consistently and significantly associated with expected satiety [i.e. the expectation that a food will stave off hunger between meals [14, 17, 18]], as well as expected satiation [i.e. the feeling of fullness a food is expected to deliver immediately after eating [16, 19, 20]]. Typically, larger portions are selected when foods are expected to be less satiating and less filling, though multiple factors can influence the direction of these relationships [e.g. familiarity, energy density and palatability [21]].

In contrast, no associations have been found between ideal portion size and age in years [20, 22]. This may be due to studies focussing on a relatively young and healthy adult population, as these studies report a mean age of < 40 years old [20, 22], and research has shown that older adults (typically ≥ 50 yrs old) tend to consume smaller meals [23]. In addition, no associations have been found between ideal portion size and BMI [14, 20, 22, 24]. In this case, it is important to note that whilst larger portions have been identified as a driver of food intake [1] and have been linked to overweight and obesity due to corresponding upwards trends overtime [9, 10, 25], ideal portion size measures are concerned with a single eating session which in and of itself may not be expected to predict BMI. This is because change in body weight occurs over a longer period of persistent positive/negative energy balance relating to energy intake and expenditure [26], and notably, portion size has similar effects on consumption irrespective of BMI in both adults and children in the context of a single meal [2, 3, 5, 6]. There is also some evidence to suggest that fat-free mass, rather than BMI, is positively associated with ad libitum food intake [27, 28]. This means that, from a theoretical perspective, measures of ideal portion size may not be expected to significantly correlate with age or BMI.

Taken together, previous research suggests that ideal portion size may be used as a ‘proxy’ measure of food intake in eating behaviour research. However, to our knowledge, current tasks cannot be used remotely as part of a web-based survey. One recent study found some evidence of agreement between a standard computer-based assessment of portion size and a ‘simplified’ portion selection task that could be used online (with 5–7 portion size images loaded into a horizontal slider and vertical Likert scale), but such tasks may be limited in terms of the variability in portion size that can be displayed to participants [29]. For instance, in a previous online study, we asked participants to scroll through a large selection of portion size photographs as part of a Likert-scale type measure, but noted that functionality and the ‘animated’ presentation of portion sizes from a computer-based task were lost, predominantly due to a need to present smaller-scale images simultaneously rather than consecutively onscreen [22]. We also note that another recent study used a screen-share service to allow some participants to complete a computer-based task to measure ideal portion size from home [18], but this method is not ideal when recruiting larger samples given that individuals will need access to (and be willing and able to use) specific video conferencing software on a one-to-one basis with a researcher.

Therefore, to pilot test the validity of an online tool as a measure of participants’ ideal portion size, this study had two main objectives:

- Objective and hypothesis 1: To test *convergent* validity of our novel online portion size selection task, this study aimed to replicate well-established relationships between ideal portion size and related drivers of food intake that have been identified in past studies using a laboratory-based computer task measure of portion size selection. As such, it was hypothesised that ideal portion size (using our online portion size selection task) would be significantly correlated with expected satiety and expected satiation.
- Objective and hypothesis 2: To test *divergent* validity of our novel online portion size selection task, this study aimed to replicate a lack of relationship between ideal portion size and relevant demographic factors that have been identified in past studies using a laboratory-based computer task measure of portion size selection. As such, it was hypothesised that ideal portion size would not be significantly correlated with age or BMI.

## Method

### Study design

Using a cross-sectional design, this online study examined associations between ideal portion size and four relevant measures (expected satiety, expected satiation, BMI, and age). Participants were directed to the study via an anonymous link to the survey software ‘Qualtrics’ (Qualtrics, Provo, UT). Judgements of foods (including ideal portion size, expected satiety, and expected satiation) were collected in response to photographs of six test foods, in three task blocks. In the first task block, participants provided ratings of liking, desire to eat, expected satiation, and familiarity for each food in turn by responding to a static photograph of a 500-kcal (kilocalories) portion of the given food. In the second and third task blocks, participants completed ideal portion size and expected satiety tasks respectively for each food, using the novel online portion size selection tool (see below for more details). Presentation order of foods within each task block was randomised using the in-built randomiser function, and questionnaire measures were presented to participants within the survey in Qualtrics. The study was completed in approximately 20 min. Study design, methods, data analysis procedures, and hypotheses were preregistered on the Open Science Framework (OSF) before data collection had begun [30].

### Participants and recruitment

Participants were recruited using ‘Prolific’, a global participant recruitment platform that circulates online research studies to its database of volunteers, who sign up to participate in paid online research studies via the platform website [for more information, see [31]]. Eligibility criteria were specified before data collection began; participants were included if they had normal or corrected-to-normal vision, and if they were 18–55 years old [in line with procedures reported in [20]]. Participants were excluded if they had dietary restrictions (i.e. a vegetarian or vegan diet, food allergies or intolerances), to ensure that realistic judgements could be given in response to test foods. Participants were also excluded if they were currently on a diet, or if they self-identified as having a current or historical diagnosis of eating disorders. Informed consent was obtained from all participants via an online form at the beginning of the survey. Before completing the consent form, participants were presented with an online information sheet and informed that the aim of the research was to ‘collect consumer beliefs about different food products’. Participants were compensated for their time with a payment of £3.13 on Prolific in accordance with platform guidelines on fair pay. The study was approved by the Department of Psychology Research Ethics Committee at Swansea University.

### Measures

#### Online portion size tool

##### Test foods

Photographs of seven test foods were presented to participants, including one food (cream and jam doughnut) that was only presented as part of a demonstration of the tool and as such was not included in data analyses (see Table 1 for macronutrient information). All test foods were selected on the basis that they would likely be familiar to participants, and were photographed from a top-down view against a plain background using a high-resolution digital camera and tripod with lateral arm. Chicken chow mein and crisps were photographed on a white dinner plate (271-mm diameter), and granola was photographed in a shallow white bowl (204-mm diameter, 36-mm depth). All other foods were photographed on a smaller white dessert plate (230-mm diameter). In line with many of the previous research studies using ideal portion size tasks [16, 17, 19, 22], each food was photographed 50 times, with each successive photograph displaying a portion that incrementally increased in size by ≈ 20 kcal. This meant that the smallest portion shown for each food was ≈ 20 kcal, and the largest ≈ 1000 kcal. Photographs were edited using Microsoft Photos for Windows 10 and PhotoScape v3.7. When uploaded to the online survey, digital dimensions for all images were 460 × 345 pixels. Food photographs used are available on the OSF [30].

**Table 1:** Test foods used for photographs in ideal portion size tool

	Kcal/100 g	Fat/100 g	Sugars/100 g	Salt/100 g
Granola	433.5	13.3	20.5	0.0
Chicken chow mein ^a^	96.0	2.5	2.4	0.6
Salted crisps (potato chips)	476.0	21.0	1.6	1.8
Madeira sponge cake	382.0	14.6	33.0	0.6
Chocolate buttons	535.0	30.0	56.0	0.2
Skittles (fruit-flavoured candy)	404.0	4.2	89.9	0.0
Cream and jam doughnut ^b^	317.4	14.4	13.2	0.4

^a^This food was low energy density [< 2.5 kcal/ g [32]]

^b^This food was only presented to participants as part of a demonstration of the portion size tool, and test responses were not included in data analyses

##### Formatting and tool set-up in ‘Qualtrics’

To create a web-based measure of portion size, we used JavaScript code [33] posted to an open source community board to adapt the slider question format in Qualtrics. This allows photographs to be loaded into a type of ‘image carousel’, whereby moving the slider from left to right changes the displayed image onscreen (for the full JavaScript code and source, see Additional file 1). Slider size was modified to have a minimum value of ‘1’ and a maximum value of ‘50’ to allow a photograph to be added for each point of the scale, grid lines and labels were removed, and a custom start position was used to set the cursor at the midpoint (≈ 500-kcal portion). Participants were required to click or drag the cursor button before they could submit a response. For each test food, 50 photographs were then loaded into the slider question in successive order, from the smallest to the largest portion size. As photographs were loaded simultaneously into the slider, with each consecutive point displaying a new photograph (and incrementally smaller or larger portion), moving the cursor of the slider generated an ‘animated’ effect by which the portion of food appeared to grow or shrink with each interaction. This visual effect appears to be comparable to that achieved using a laboratory-based computer task to measure ideal portion size [see previous description in the introduction above [16]].

To use the measure, participants were instructed to click or drag the cursor to the left of the scale to decrease the portion displayed, and to click or drag the cursor to the right of the scale to increase the portion displayed. For each test food, participants were instructed to ‘select your ideal portion size to eat right now’, or to select the portion that they would ‘need to eat right now in order to prevent hunger until your next meal’ to measure expected satiety. The name of the presented food was included in the question. To help mitigate the potential influence of the starting portion size and encourage participants to view a range of portions before selecting a response, participants first practised using the portion size tool with a dummy test food (cream and jam doughnut) by slowly dragging the cursor to the far left and far right anchors of the scale; they were only able to continue with the study once they had successfully completed the demonstration. The point at which participants set the cursor was automatically recorded by Qualtrics; this could be used to identify the selected photograph number, corresponding weight of food displayed (in g/grams), and the portion size selected (in kcal) for each food. A video demonstration of this web-based tool can be viewed on the OSF [30].

#### Food ratings

To provide food ratings, participants responded to a photograph of the median portion size for each test food (≈ 500 kcal portion), using a series of 100mm visual analogue scales with the anchors ‘Not at all’ to ‘Extremely’. Participants were asked to provide ratings of expected satiation (‘how full would you expect to feel after eating the portion of food displayed above?’), liking (‘how much do you like the taste of this food?’), desire to eat (‘how much would you like to eat this food right now?’), and familiarity (‘how familiar is this food?’). Participants also rated their baseline hunger (‘how hungry do you feel right now?’) and baseline fullness (‘how full do you feel right now?’). Whilst providing food ratings, participants responded to two additional questions as attention checks (on both occasions, participants were asked to ‘drag the slider all the way to the left’).

#### Questionnaires

Following a similar approach to past studies [34, 35], the three-factor eating questionnaire-R18 [TFEQ-R18 [36];] was used to characterise the overall sample and assess dietary restraint, uncontrolled eating, and emotional eating traits. Responses were recorded using a 4-point Likert scale (e.g. definitely false/mostly false/mostly true/definitely true), and 4 items were reverse-scored. For each subscale, the sum of coded items was calculated, and raw scores were converted to a 0–100 scale (((raw score − lowest possible raw score)/possible raw score range) × 100). Higher subscale scores suggest greater tendencies for dietary restraint, uncontrolled eating, and emotional eating.

Participants were also asked to provide demographic information including their age, gender, country of residence, and time since last eating. Participants self-reported their height and weight using dropdown lists to enable calculations of BMI (kg/m^2^). To evaluate potential demand awareness at the end of the study, participants had an opportunity to explain their beliefs about the aim of the study using an open-text field, before viewing a debrief form.

To avoid influencing responses to foods, participants completed the TFEQ-R18 and self-reported height and weight after completing main task blocks.

### Validity

To test convergent validity of the portion size selection tool, participants’ selected ideal portion size (in kcal) was compared to their selected portion size for expected satiety (in kcal) and rating of expected satiation (100-mm VAS) for foods. To test divergent validity of the portion size selection tool, participants’ selected ideal portion size (in kcal) was compared to their self-reported age (in years) and BMI (kg/m^2^).

### Sample size

Using the software programme ‘G*Power v.3.1.9.7’, it was estimated that 42 participants were required to detect a correlation *ρ* of 0.50 (1−*β* = 0.80, *p* = .01, two-tailed), as previous research suggests that expected satiety and expected satiation are ‘moderately’ associated with ideal portion size [14, 16–18]. Data collection was stopped when 56 responses had been recorded to account for unusable data (e.g. incomplete responses, multiple responses from the same participant ID). After checking inclusion and exclusion criteria, 49 responses were complete and eligible for the study.

### Data analysis

For main data analyses, ideal portion size and expected satiety ratings were converted to kcal, and all ratings were collapsed across foods by calculating the mean. A Shapiro-Wilk test showed that data for age (*p* < .001), BMI (*p* = .001), ideal portion size (*p* = .008), and expected satiety (*p* = .002) were not normally distributed. As log transformation did not correct the data, an appropriate non-parametric test was used to calculate coefficients in a bivariate correlation matrix (Spearman’s Rho). This was used to test the hypotheses that ideal portion size would be significantly correlated with expected satiety and expected satiation, and would not be significantly correlated with age or BMI.

No participant failed both attention checks (to warrant exclusion from the study); however, 4 participants failed a single attention check. Outliers were checked for additional food ratings (as factors that may influence ideal portion size), as well as ideal portion size and main predictors of interest (expected satiety, expected satiation, age and BMI). Outliers were removed listwise or pairwise from data analyses accordingly (1.5 × IQR). Identified outliers included a single participant that was removed from all data analyses, as they had a mean food liking score of 7.2. Identified outliers also included three participants who were removed from pairwise analyses; one participant self-reported a BMI of > 40.0 kg/m^2^, and two participants had a mean ideal portion size of 634.6 kcal and 737.1 kcal respectively. This meant that data from 48 participants were included in data analyses. Significance was determined using the standard *p* < .05. Data were analysed using IBM SPSS v26.

To check that associations between ideal portion size and relevant measures of interest (expected satiety, expected satiation, age, BMI) were robust, non-parametric (Spearman’s Rho) partial correlation coefficients were calculated to account for effects of baseline hunger and fullness, and the main analysis was repeated with individual test foods to calculate separate coefficients (following the same procedure as above). For the latter, significance was determined using the Bonferroni-corrected *p* = .008. A Bayesian non-parametric correlation matrix (Kendall’s tau-b) was used to explore strength of evidence for associations in the main analysis. Bayes factors were interpreted using the descriptors ‘anecdotal’, ‘substantial’, ‘strong’, and ‘very strong’, to indicate support for alternative and null hypotheses [37], and 95% credible intervals (CI) are reported. Bayesian analyses were conducted using the open-source software JASP v.0.11.1.0, with a default prior setting of 1.

## Results

### Participant characteristics

Participants included 24 females and 24 males. Most participants self-reported a country of residence in Europe (*N* = 37), 5 participants South America, 3 participants North America, and 3 participants South Africa. For the overall sample, mean scores on subscales of the TFEQ-R18 suggest trait levels of dietary restraint, uncontrolled eating, and emotional eating were comparable to past studies from our laboratory [34, 35, 38]. When asked to report beliefs about the aim of the study, 7 participants mentioned an interest in portion size, but no participants appeared to comment on the relationship between portion size and predictor variables (expected satiety, expected satiation, age, and BMI) specifically. See Table 2 for all other participant characteristics, and see Table 3 for descriptive statistics for food ratings.

**Table 2:** Sample characteristics (*N =* 48)

Demographics	Range	M (SD)
Age (years)	18.0–55.0	29.0 (10.7)
BMI (kg/m^2^)	17.7–34.4	23.5 (4.4)
Baseline hunger (100mm)	0.0–100.0	33.0 (31.3)
Baseline fullness (100mm)	0.0–94.0	52.5 (28.0)
Time since eating (min)	0.0–943.0	173.7 (205.7)
Restraint^1^	0.0–77.8	38.2 (21.5)
Uncontrolled eating^1^	3.7–85.2	42.0 (19.5)
Emotional eating^1^	0.0–100.0	43.7 (34.5)

^1^Subscale score of the TFEQ-R18, reported on a 0–100 scale

**Table 3:** Descriptive statistics for food ratings, ideal portion size, expected satiety, and expected satiation. Mean (SD) is reported

Variable	Collapsed across foods^a^	Granola	Chicken chow mein	Salted crisps	Madeira sponge cake	Chocolate buttons	Skittles
Food liking (100mm)	67.6 (12.3)	58.6 (31.4)	68.7 (27.9)	70.9 (26.8)	63.1 (24.7)	76.7 (24.2)	67.4 (25.8)
Food familiarity (100mm)	70.4 (17.3)	70.8 (28.2)	66.2 (29.3)	74.6 (26.9)	60.3 (31.8)	74.9 (28.9)	75.5 (30.1)
Desire to eat (100mm)	43.7 (17.2)	30.7 (30.9)	46.6 (35.9)	45.6 (31.8)	44.5 (32.9)	52.9 (34.9)	41.9 (30.7)
Expected satiation (100mm)	53.9 (17.5)	68.9 (28.2)	86.0 (13.9)	54.8 (29.5)	41.4 (27.8)	40.4 (30.0)	32.0 (29.7)
Expected satiety (kcal)	460.3 (233.7)	363.4 (205.5)	392.7 (266.9)	405.7 (251.4)	599.6 (289.8)	459.1 (271.9)	541.6 (344.2)
Ideal portion size (kcal)	281.7 (133.5)	239.8 (156.5)	316.6 (235.0)	266.1 (237.5)	462.3 (292.6)	256.1 (211.9)	250.4 (269.2)

^a^Collapsed across foods by averaging scores for individual items

### Associations between ideal portion size and measures relevant to food intake

When collapsed across foods, there was a significant positive association between ideal portion size and expected satiety (*r*_s_(44) = .480, *p* = .001). There were no significant associations between ideal portion size and expected satiation (*r*_s_(44) = −.287, *p* = .053), age (*r*_s_(44) = −.032, *p* = .835), or BMI (*r*_s_(43) = −.111, *p* = .468). However, after controlling for effects of baseline hunger and fullness, there was a significant, negative association between ideal portion size and expected satiation, whereby a larger ideal portion size was selected when expected satiation was reduced (*r*_s_(42) = −.310, *p* = .041). All other results were unchanged.

Analyses with individual foods showed similar results. For all foods, there was a significant positive association between ideal portion size and expected satiety (with the exception of granola), and there were no significant associations between ideal portion size and expected satiation, age, or BMI. However, after controlling for effects of baseline hunger and fullness and correcting for multiple comparisons, only associations for salted crisps, Madeira sponge cake, and Skittles remained significant (between ideal portion size and expected satiety). See Table 4 for correlations with individual foods.

**Table 4:** Correlations (*r*_s_) between ideal portion size and predictors of food intake, for individual test foods

Predictor variable	Granola	Chicken chow mein	Salted crisps	Madeira sponge cake	Chocolate buttons	Skittles
Age (years)	−.317 (−.313)	.047 (.077)	.270 (.274)	−.014 (−.013)	−.025 (.004)	−.129 (−.138)
BMI (kg/m^2^)	.008 (.014)	.106 (.172)	.106 (.114)	−.051 (−.036)	−.002 (.028)	.169 (.171)
Expected satiation (100mm)	−.004 (−.022)	.158 (.116)	−.032 (−.030)	−.247 (−.229)	−.211 (−.224)	−.116 (−.101)
Expected satiety (kcal)	.237 (.233)	.391* (.321)	.461* (.459)*	.508** (.498)**	.393* (.380)	.472* (.469)*

Coefficients accounting for effects of baseline hunger and fullness are given in brackets

**Correlation is significant, *p* < 0.001; *correlation is significant, *p* < 0.008 (Bonferroni-correction applied)

Bayesian analyses showed that, when collapsed across foods, there was ‘substantial’ evidence in favour of no association between ideal portion size and age (BF_10_ = 0.196, 95% CI −0.212, 0.168), and BMI (BF_10_ = 0.277, 95% CI −0.277, 0.108). There was ‘anecdotal’ evidence in favour of a significant association between ideal portion size and expected satiation (BF_10_ = 1.591, 95% CI −0.391, −0.010), and ‘very strong’ evidence in favour of a significant association between ideal portion size and expected satiety (BF_10_ = 50.172, 95% CI: 0.132, 0.511).

Bayesian analyses also showed that for all individual foods, there was ‘very strong’ and ‘strong’ evidence in favour of a significant association between ideal portion size and expected satiety (with the exception of granola), and substantial evidence in favour of no association between ideal portion size and BMI (with the exception of Skittles). Results also appeared to favour no association between ideal portion size and expected satiation, and ideal portion size and age, though there were some differences in the strength of evidence between foods. See Table 5 for Bayesian analyses with individual foods.

**Table 5:** Bayes factors (BF_10_) for correlations between ideal portion size and predictors of food intake, for individual test foods

Predictor variable	Granola	Chicken chow mein	Salted crisps	Madeira sponge cake	Chocolate buttons	Skittles
Age (years)	2.00 (−0.40, −0.02)	0.20* (−0.16, 0.21)	0.98 (−0.01, 0.36)	0.19* (−0.19, 0.19)	0.21* (−0.23, 0.15)	0.30* (−0.30, 0.12)
BMI (kg/m^2^)​	0.19* (−0.18, 0.19)	0.24* (−0.12, 0.25)	0.24* (−0.13, 0.25)	0.20* (−0.23, 0.15)	0.19* (−0.20, 0.19)	0.35 (−0.10, 0.31)
Expected satiation (100mm)	0.19* (−0.19, 0.20)	0.35 (−0.08, 0.29)	0.19* (−0.20, 0.17)	0.84 (−0.35, 0.02)	0.45 (−0.32, 0.06)	0.24* (−0.27, 0.15)
Expected satiety (kcal)	0.83 (−0.02, 0.35)	13.74** (0.09, 0.46)	68.14*** (0.14, 0.51)	191.36*** (0.17, 0.54)	10.17** (0.08, 0.46)	28.10** (0.12, 0.52)

Bayes factors shown are for non-parametric correlations (Kendall’s tau) that do not account for effects of baseline hunger and fullness

Bayes factor indicates *** ‘very strong evidence’; ** ‘strong evidence’; * ‘substantial evidence’; all other factors indicate ‘anecdotal’ or ‘no evidence’. Bayes factor > 1 indicates evidence in favour of an association (H1 over H0)

95% CI are given in brackets

## Discussion

This pilot study provides preliminary evidence for the validation of a Qualtrics-based portion size selection tool to measure ideal portion size. As predicted, ideal portion size across foods significantly correlated with expected satiety and did not significantly correlate with age or BMI. There was also some evidence in favour of an association between ideal portion size and expected satiation, but this was weaker and less consistent compared to expected satiety. A similar pattern of results was found for individual foods, and results are generally comparable to those found in past studies using laboratory-based computer assessments of ideal portion size. First, moderate associations (*r* > 0.50) have been observed for expected satiety [14, 17, 18] and expected satiation [16] in previous research, and given findings of a consistent association between ideal portion size and expected satiety in the present study, these results provide partial support for the convergent validity of the current measure. Second, very weak associations (*r* < 0.12) have previously been observed for age [22] and BMI [14, 22], and as evidence also favoured no significant associations with ideal portion size in this study, results provide support for divergent validity of the current measure.

It is important to acknowledge that the association between ideal portion size and expected satiety differed to that of expected satiation in this study, despite the previous suggestion that expected satiety and expected satiation are highly correlated [11]. This may be explained by the method used to measure expected satiation. First, in line with previous research [14, 16, 18, 19, 22, 24], we chose to use the median portion size produced for each food (500-kcal) as the reference for participants to provide food ratings. This was likely larger than the typical portion size consumed by participants for each food, potentially biasing the relationship between expected satiation and ideal portion size. Second, though use of visual analogue scales to measure expected satiation has been validated with reference to food intake [21], it has been suggested that the meaning of maximum intensity anchors (e.g. ‘not at all – extremely’) can differ across individuals, and obscure variances in scores [39, 40]. The ‘general labelled magnitude scale’ (gLMS) has been proposed as a stronger approach, as it includes intermittent labels and additional points beyond maximum descriptors to improve sensitivity of responding (e.g. ranging from ‘barely detectable’ to ‘strongest imaginable sensation of any kind’) [40]. These scales have been used previously to measure satiation in both clinical and non-clinical groups [41, 42], and warrants further investigation for the measurement of expected satiation in an online setting, particularly when examining between-group differences where variance across individuals is of particular interest.

It should also be highlighted that studies using a psychophysical measurement of expected satiation have reported stronger associations with ideal portion size [for a discussion, see [21]]. In our study, changing the question used to frame the online portion size task seemingly allowed us to measure expected satiety with greater success, and there is scope to further develop the complexity of the ideal portion size tool to also allow assessment of expected satiation. For example, to implement a ‘matched fullness task’ [16, 19], a fixed-portion for a ‘standard’ food may be placed to the left of a ‘comparison’ food within the portion size tool by combining images into a single file to load on each point of the scale (meaning two foods are simultaneously presented onscreen as opposed to a single food in this study). The image of the standard can then be kept the same at each point, whilst the image of the comparison food can be varied, allowing participants to select the portion of the comparison food that would leave them ‘feeling equally full’. This then allows for measurement of expected satiation for the standard food whilst controlling for potential effects of differences across foods in terms of volume, weight, and energy density [21]. However, in an online setting, the potential trade-off between study length/ complexity and data quality should be considered [43, 44]. Compared to collecting VAS measures, such tasks will require set-up of multiple additional task blocks to complete within surveys and may also require participants to have access to specific devices to display tasks correctly. Indeed, further research is needed into how factors such as screen size, resolution, and orientation influence the validity of photograph-based assessments of portion size in an online setting.

In the present study, the association between expected satiation and ideal portion size across foods also differed depending on the statistical control of hunger and fullness at baseline. Given that there was little evidence of a significant association between expected satiation and ideal portion size for individual foods, and Bayesian analyses also found some substantial evidence in favour of no association, we would conclude that there is little to no evidence of an association between expected satiation and ideal portion size in this study (see discussion of limitations for the measure of expected satiation used in this study above). However, from a theoretical perspective, it is important to consider effects of baseline hunger and fullness as factors that can influence eating-related outcomes. Indeed, ratings of current hunger and fullness have been significantly associated with ideal portion size in previous research [22, 24]. Controlling for participant differences in appetite at baseline is also recognised to be best practice in laboratory studies measuring food intake, usually by asking participants to consume a standardised meal in the laboratory and/ or abstain from eating for a specific period before the main study [45]. As such protocols can be difficult to implement in online research, collecting ratings of current hunger and fullness as potential covariates is one way to account for these effects.

Taken together, the results of this pilot study highlight the potential of using web-based survey software to develop a dynamic, photograph-based tool to measure aspects of eating relating to meal size and consumption. Compared to our previous attempt to measure ideal portion size within an online survey [22], this tool appears to have greater success in preserving key elements of laboratory-based computer tasks; (1) images are presented consecutively when participants interact with stimuli, giving rise to an ‘animated’ change in portion size, and (2) as a single photograph is displayed on screen at any time, images can be relatively large in size, allowing participants to perceive smaller changes between consecutive portions. As a ‘simplified’ continuous-scale measure of portion size, this tool may have particular implications for the assessment of self-reported food intake in wider research, given that it consists of fewer trials and is less effortful compared to previous tasks.

This tool has some specific practical advantages for use in future research. As identified for laboratory-based computer assessments of ideal portion size [14], a photograph-based measure can remove barriers associated with testing effects on ad libitum food intake (e.g. relating to products being discontinued or becoming difficult to source, time needed to prepare foods in advance of multiple test sessions, and food waste). In addition, online testing is known to significantly reduce the time needed for data collection, meaning larger samples are more likely to be viable (e.g. participation is no longer restricted by a need to arrange one-to-one timeslots at a specified location, participants are free to take part at their own convenience, and multiple participants may complete the study in parallel). As paradigms consisting of many trials can often be difficult to implement for online research, the use of this tool may be considered a more accessible, alternative approach to developing such a task; Qualtrics has an intuitive user interface, allowing for the seamless integration of the tool into a large-scale online survey with additional questions of multiple formats, and this approach does not require researchers to have an advanced understanding of coding language.

There are some limitations of this study that should be acknowledged. Internal consistency between questionnaires and ratings of foods was not measured, as we were interested in piloting single items that can be included within large-scale online surveys that are not necessarily expected to relate to each other. Test re-test reliability was not measured, as to our knowledge, there is little evidence to support or theoretical justification for the notion that ideal portion size is a stable trait measured over time. Indeed, expectations for satiety and satiation, as identified drivers of ideal portion size, are believed to be learned and influenced by external cues in the eating environment [21]. This issue of stability is particularly pertinent for online studies, as they traditionally lack constraints used to increase experimental control in laboratory studies that seek to minimise extraneous influences on ideal portion size when assessed at different times or between different groups, such as the influence of current appetite. However, as this pilot study was limited to an online-only setting, future research is needed to validate this tool with direct comparisons to laboratory-based computer assessments, as well as actual food intake.

The use of this tool may also be limited by the type of food used. In this study, exploratory analyses showed that ideal portion size failed to correlate with both expected satiety and expected satiation for granola. Though there are likely to be some cultural differences, one explanation for this is that cereals are consumed as ‘breakfast foods’ in many countries [46–53], and regularly consuming foods during a specific mealtime (at the exclusion of other mealtimes) may increase the likelihood that individuals will select a ‘habitual’ rather than an ‘ideal’ portion size for some foods [17]. For instance, though not significantly different, McLeod et al. [18] recently reported that the association between ideal portion size and expected satiety was weaker when ‘breakfast foods’ were presented at lunch compared to breakfast time, suggesting that ideal portions may be driven less by *current* expectations for satiety outside of the usual eating context and more by *learned* expectations associated with the usual eating context. Given that time of participation can be difficult to control in online studies, it may be of interest to consider whether potential test foods may be associated with specific meal contexts in future research.

When considering future applications of this tool, it is also important to consider how well participants will be able to judge chosen test foods in photographs, particularly when using this ideal portion size tool as a proxy measure of food intake. As mentioned previously, screen size is one factor that might influence the perceived size of foods and portions, and researchers should consider using methods that help control for differences in screen size across participants (e.g. calibration tests to record screen size, and the inclusion of common objects in photographs to assist perception of portion size). It should be noted that some foods are more difficult to photograph than others (e.g. the ability to distinguish encased fillings or layers in a food is often lost when photographing stimuli from a top-down view in order to show changes in portion size). For such foods, it may be unsuitable to use the tool as presented here, and researchers may need to consider additional adaptations before use (e.g. presenting images of foods from multiple angles, including a cross-section of the centre of the food item). Consideration should also be given to whether or not the experience of the food would align with expectations derived from photographs. For example, when based on appearance alone, it can be difficult for participants to perceive differences in flavour and texture that they would otherwise perceive when tasting a food. This means that highly familiar products are likely to be most appropriate for use, and this tool may need to be accompanied by additional information if more novel foods are to be used in future research (e.g. text-based sensory descriptions).

## Conclusions

Overall, data from this pilot study support convergent and divergent validity of a web-based portion size selection tool to measure ideal portion size. Results suggest that this measure may be used to assess decisions focussed on meal size prior to eating, and as an indicator of food intake. This has wider implications for use in online research studies concerned with eating behaviour. However, given the relatively small scale of this pilot study, and limitations around the assessment of validity in an online-only setting, further research is needed to assess efficacy as it relates to actual consumption.

## Supplementary Information

**Additional file 1..** Online supplementary material

## Data Availability

The datasets generated and analysed during the current study are available in the Open Science Framework repository, https://osf.io/yq9fk/. Project DOI: 10.17605/OSF.IO/YQ9FK.

## Abbreviations

BMI: Body mass index (kg/m^2^)
OSF: Open Science Framework
TFEQ-R18: Three factor eating questionnaire-R18
95% CI: 95% credible interval
