# Supplementary material for: Testing an online measure of portion size selection: a pilot study concerned with the measurement of ideal portion size
Source: Pilot Feasibility Stud. 2021 Sep 17;7:177. doi: 10.1186/s40814-021-00908-x (PMC8446476; doi:10.1186/s40814-021-00908-x)
Supplement: Supplementary file 1 — Additional file 1.. Online supplementary material [file 40814_2021_908_MOESM1_ESM.docx]

**Online supplementary material**

**Testing an online measure of portion size selection: A pilot study concerned with the measurement of ideal portion size.**

Rochelle Embling^1^, Menna Price^1^, Michelle Lee^1^, Laura L. Wilkinson^1^

^1^Department of Psychology, College of Human and Health Sciences, Swansea University, Swansea, SA2 8PP, UK. Email RE: 825379@swansea.ac.uk

Corresponding author: Rochelle Embling, Department of Psychology, College of Human and Health Sciences, Swansea University, Swansea, SA2 8PP, UK. Phone: 01792 29 5279. Email: [rochelle.embling@outlook.com](mailto:rochelle.embling@outlook.com).

**Description of supplementary materials:**

**Supplementary material 1** JavaScript code used, and original source cited.

**OSF project DOI:** 10.17605/OSF.IO/YQ9FK

**Availability of data and materials:** Data described in this article is freely and publicly available on the Open Science Framework (<https://osf.io/>). Project DOI: 10.17605/OSF.IO/YQ9FK.

**Online Supplementary Material**

**Supplementary material 1** JavaScript code used, and original source cited.

**Source:**

Sunami, N. (https://www. qualtrics. com/community/profile/activity/Nami). (2019). *How can I create a personalized graphic slider Question with JavaScript? (Discussion board)*. Qualtrics Community. https://www.qualtrics.com/community/discussion/6560/how-can-i-create-a-personalized-graphic-slider-question-with-javascript

Qualtrics.SurveyEngine.addOnReady(function()

{

/*Place your JavaScript here to run when the page is fully displayed*/

// Assign the image on the HTML based on the image id. #myImage should correspond with the `id=` parameter.

var img = jQuery("#myImage");

currentQID = this.questionId

// Function to look for track changes in slider

// code inspired by https://www.qualtrics.com/community/discussion/5963/dynamic-accessing-slider-value

jQuery("input[type = hidden]").change(function() {

// get the current position of the slider and assign it to `answer`

var answer = parseInt(jQuery("#" + currentQID + " input.ResultsInput").eq(0).val());

// print out the answer to the browser console

console.log(answer) ;

// Create the list of Images 1-50 -- in order of presentation. Can be expanded or reduced according to the length of the slider question.

var images_list = [

'Link to image 1 in Qualtrics Library',

'Link to image 2 in Qualtrics Library',

'Link to image 3 in Qualtrics Library',

'Link to image 4 in Qualtrics Library',

'Link to image 5 in Qualtrics Library',

…

'Link to image 50 in Qualtrics Library',

]

// Swap the image according to the slider answer, so that the slider position 1 corresponds with the first image (zero indexed).

img[0].src = images_list[answer - 1]

});

});

//To be entered in html view of image slider//

<div style="text-align: center;"><img id="myImage" src="link to image 25 in Qualtrics library for median portion size" style="width: 460px; height: 345px;" /></div>
